# THE IMPACT OF PARTICIPATION IN SOCIAL HEALTH NETWORKS

**DOI:** 10.1093/geroni/igz038.2449

**Published:** 2019-11-08

**Authors:** Anna Faul, Mona Huff, Samantha G Cotton, Pamela Yankeelov, Joe D’Ambrosio, Megan Austin

**Affiliations:** University of Louisville, Louisville, Kentucky, United States

## Abstract

Stress and compassionate fatigue are common among graduate level students working in healthcare professions, however, few studies focus on preventative self-care and its’ impact on these learners. As part the University of Louisville’s Behavioral Health Workforce Enhancement Training Program (BHWET), graduate students are trained to work with older adults in rural communities. The focus of our BHWET program is to provide holistic, behavioral health care through our FlourishCare Network. As part of the student’s weekly curriculum, an interdisciplinary group of learners from counseling psychology, social work and psychiatric nursing were invited to the attend the sessions were invited to participate in a 2-semester Microclinics and Health Matters course that was designed to promote self-care and harness the power of social networks to promote health. A total of 15 students completed the program. Biomarkers including BMI, Cholesterol, A1C, Blood Pressure were taken every week time the course was offered. Cortisol levels were taken every 4 months to measure stress levels. Across the initial 12 weeks of programming, there were positive outcomes for the participants in terms of either maintenance of healthy goals or biomarkers. Additionally, the program had an impact on the older adult clients that were being served by the students compared to students that did not participant. In a review of the plan of care items, which is central to our work with FlourishCare clients, plan of care items showed a stronger focus on connecting clients to social health interventions and a stronger connection to education about health-related content.

